# Vancomycin heteroresistance in coagulase negative *Staphylococcus* blood stream infections from patients of intensive care units in Mansoura University Hospitals, Egypt

**DOI:** 10.1186/s12941-017-0238-5

**Published:** 2017-09-19

**Authors:** Ghada El-Saeed Mashaly, Rasha Hassan El-Mahdy

**Affiliations:** Department of Medical Microbiology and Immunology, Faculty of Medicine, Mansoura University, Mansoura, 35516 Egypt

**Keywords:** Heteroresistance, Vancomycin, CoNS, ICU

## Abstract

**Background:**

Vancomycin heteroresistance in coagulase negative *Staphylococci* (CoNS) is a recent health concern especially in serious infections like bloodstream infections as it may lead to failure of therapy. Little information is available about the prevalence vancomycin heteroresistance in CoNS causing bloodstream infections in intensive care units (ICUs) patients of Mansoura University Hospitals (MUHs).

**Methods:**

This prospective study enrolled 743 blood samples collected from ICUs patients presented with clinical manifestations of bloodstream infections over the period extending from January 2014 to March 2016. Samples were processed, coagulase negative *Staphylococci* were identified by routine microbiological methods and the absence of coagulase activity. Species were identified by API Staph 32. Oxacillin resistant CoNS were identified by cefoxitin disc diffusion method. Susceptibility testing of isolated CoNS to vancomycin was carried out using vancomycin agar dilution method. *Mec A* gene detection by PCR was done for oxacillin resistant isolates. Screening for vancomycin heteroresistance was done on brain heart infusion (BHI) agar containing 4 μg/mL vancomycin. Confirmation of vancomycin heteroresistance was carried out by population analysis profile (PAP).

**Results:**

A total of 58 isolates were identified as CoNS from patients of clinically suspected bloodstream infections. The identified species were 33 (56.9%) *Staphylococcus epidermidis*, 12 (20.7%) *Staphylococcus capitis*, 7 (12.1%) *Staphylococcus haemolyticus*, and 3 isolates (5.2%) *Staphylococcus lugdunesis*. Three isolates were unidentified by API Staph 32. Forty-four (75.9%) isolates were oxacillin resistant. *Mec A* gene was detected in all oxacillin resistant isolates. All isolates had susceptible vancomycin MICs by agar dilution. Nine isolates (15.5%) could grow on BHI agar containing 4 μg/mL vancomycin. These isolates showed heterogeneous profile of resistance to vancomycin by population analysis profile.

**Conclusions:**

Vancomycin heteroresistant CoNS causing bloodstream infections is growing unrecognized health hazard in ICUs patients. These isolates have susceptible vancomycin MICs. Screening methods are recommended and should be considered to improve clinical outcome in these high risk patients.

## Background

Coagulase-negative *Staphylococci* (CoNS) are considered as a major cause of bloodstream infections (BSIs) in intensive care units (ICUs) patients [1]. Glycopeptides especially vancomycin are frequently considered as the first choice for treatment of serious infections caused by CoNS due to rise in resistance to Beta lactam antibiotics [2].

Although, glycopeptides resistance in CoNS has been first detected in 1987 [3]. The increased use of glycopeptides leads to diminished glycopeptides susceptibility among CoNS [4]. Later, another type of resistance named heteroresistance has been also described in clinical CoNS isolates in which vancomycin-intermediate subpopulation of cells exist in susceptible microbial population [5, 6].

Although, there is decrease susceptibility to glycopeptides in CoNS [6, 7], little studies were conducted on heteroresistance to glycopeptides in CoNS in comparison to *S. aureus* [8–10]. The objective of the present study was to determine the prevalence of heteroresistance vancomycin coagulase negative *Staphylococci* among patients with BSIs in ICUs of Mansoura university hospitals.

## Patients and methods

This study was conducted on patients who had BSI in the ICUs of Mansoura university hospitals from January 2014 to March 2016. Nine ICUs were included in this study; five medical and four surgical ICUs. Blood samples were collected from patients admitted to these ICUs and presented with symptoms with suspected bloodstream infections according to CDC criteria [11]. Blood stream infections caused by CoNS are considered only if at least two blood cultures positive for CoNS were collected within 5 days [12]. *Staphylococci* were identified by standard microbiological methods [13]. CONS was confirmed by negative coagulase (*coa*) gene [14]. Identification of Species was done by API Staph 32 (bioMérieux) according to the manufacture instructions.

The data including: sex, age, clinical diagnosis and systemic antimicrobial therapy were retrieved from the medical records.

### Oxacillin susceptibility testing

Resistance to oxacillin was evaluated by cefoxitin (30 µg) (Mast Diagnostics, Merseyside-UK) disc diffusion method according to the Clinical and Laboratory Standards Institute (CLSI) criteria [15]. Oxacillin resistant CoNS isolates were tested for the presence of *mecA* gene. The PCR for *mecA* gene was done as previously described [16].

### Vancomycin susceptibility testing

Agar dilution method was done to determine the vancomycin susceptibility according to CLSI guidelines [15]. Standard vancomycin susceptible *S. aureus* ATCC 29213 was included as quality control.

Isolated strains were evaluated for vancomycin heteroresistance using the brain heart infusion (BHI) screen agar method as formerly described [17]. Briefly, bacterial suspension of 0.5 McFarland turbidity was prepared from an overnight culture on blood agar plate. Four 10-µL drops of the suspension were dropped onto a BHI agar plate (Mast Diagnostics, Merseyside-UK) containing 4 µg/mL vancomycin (Sigma Chemical Co., St. Louis, Mo.). Plates were incubated at 35 °C ± 2 for 48 h; the number of colonies in each drop was counted. A strain was considered to be heteroresistant to vancomycin if ≥ 1 droplet had ≥ 2 colonies.

### Population analysis profile (PAP) for vancomycin heteroresistance

The PAP method was used to confirm vancomycin heteroresistance in subpopulations. All isolates that grown on BHI screen agar plate were included in PAP experiments [18]. Inoculums of (100 μL) 0.5 McFarland turbidity bacterial suspension and serial tenfold dilutions were cultured on BHI agar plates containing vancomycin at the concentrations 2, 4, 6, 8, 10, 12, 14 and 16 μg/mL at 35 °C. After 48 h, the number of colonies on plate in each concentration of drug was counted. Each strain was tested twice.

Heteroresistant strain was defined as any CoNS strain that has a susceptible vancomycin MIC by agar dilution method, can grow on BHI with 4 µg/mL vancomycin and has by PAP susceptibility testing a MIC greater than or equal to 8 µg/mL in the two experiments [19].

## Results

**Fig. 1:**
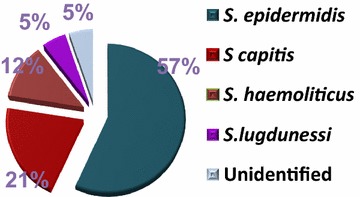
Distribution of CoNS species

Total of 743 of clinically suspected blood stream infections were involved in this study. Fifty-eight isolates of CoNS were detected by absence of *coA* gene. *S. epidermidis* was the most common isolated species. Distribution of CoNS was summarized in Fig. 1.

Resistance rates of the isolated CoNS to oxacillin were reported to be 76.6% (36/47). All isolates were categorized as vancomycin susceptible using agar dilution method according to the CLSI guidelines [15]. The MICs of vancomycin range from 0.25 to 2 and 0.25 to 1 µg/mL in oxacillin resistant and oxacillin sensitive isolates respectively. Vancomycin MICs by agar dilution method among different CoNS species are shown in Table 1.

**Table 1:** Vancomycin MICs by agar dilution and species distribution of 58 CoNS strains isolated from blood stream infections

CoNS species	Vancomycin MICs μg/ml					
	0.25	0.5	1	2	4	8
Oxacillin resistant isolates	1	12	17	14	–	–
*S. epidermidis*	–	7	10	9	–	–
*S. capitis*	1	2	3	4	–	–
*S. haemolyticus*	–	2	3	–	–	–
*S. lugdunesis*	–	–	–	1	–	–
Unidentified species	–	1	1	–	–	–
Oxacillin sensitive isolates	6	4	4	–	–	–
*S. epidermidis*	4	2	1	–	–	–
*S. capitis*	1	1	–	–	–	–
*S. haemolyticus*	1	1	–	–	–	–
*S. lugdunesis*	–	–	2	–	–	–
Unidentified species	–	–	1	–	–	–
Total	7	16	21	14	–	–

**Fig. 2:**
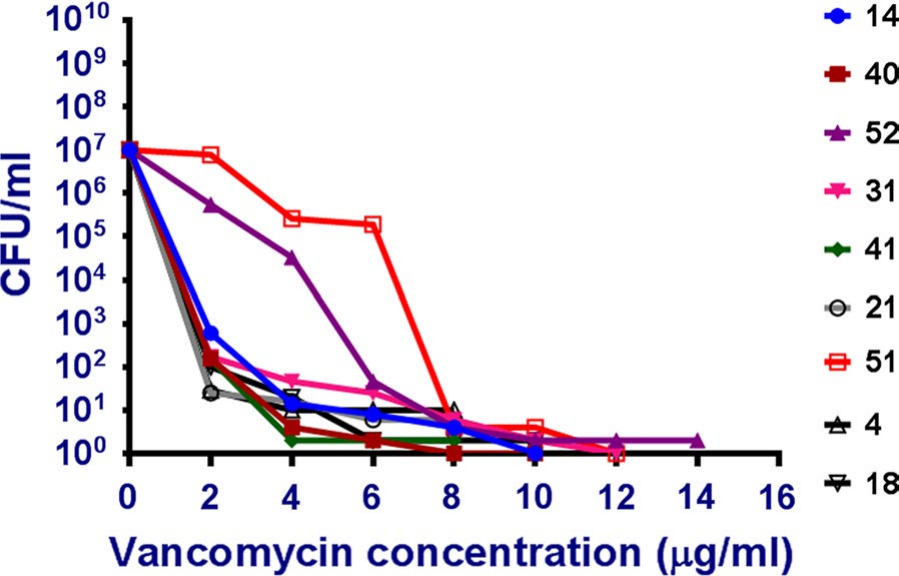
PAP analysis of nine strains can grow on BHI with 4 µg/mL Vancomycin

Nine of these CoNS isolates can grow on the BHI agar containing 4 μg/mL of vancomycin. vancomycin MICs ranged from (1–2 µg/mL) by agar dilution method. By PAP these nine isolates were in the vancomycin intermediate susceptibility zone ranging from 10 to 16 µg/mL. All these heteroresistant isolates were oxacillin resistant. Majority of heteroresistant strains were *S. epidermidis* (66.7%). The PAPs of these nine strains are shown in Fig. 2.

Most of heteroresistant isolates were isolated from patients with previous vancomycin exposure (6/9). The characteristics of the vancomycin heteroresistant CoNS isolates and Characteristics of patients are shown in Table 2.

**Table 2:** Characteristics of Vancomycin heteroresistant CoNS isolates and patients features

Strain No	*Staphylococus* species	OX	VA MIC	PAP MIC	Age (yrs)	Sex	ICU	Underlying disease	VA exposure
14	*S. epidermidis*	R	2	16	68	M	MICU	Hypertensive encephalopathy	Yes
40	*S. epidermidis*	R	2	12	59	M	MICU	Liver cirrhosis	NO
52	*S. epidermidis*	R	1	14	62	F	MICU	Pneumonia	Yes
31	*S. epidermidis*	R	2	12	66	M	SICU	Cancer colon	No
41	*S. epidermidis*	R	1	10	38	F	SICU	Motor car accident	Yes
21	*S. epidermidis*	R	2	10	25	M	SICU	Motor car accident	Yes
51	*S capitis*	R	2	14	56	M	SICU	Liver recipient	Yes
4	*S. capitis*	R	2	14	67	F	MICU	Cerebral stroke	Yes
18	*S. haemolyticus*	R	4	12	69	M	MICU	Diabetic ketoacidosis	No

*R* resistant, *Va* vancomycin, *SICU* surgical ICU, *MICU* medical ICU, *M* male, *F* female

## Discussion

Coagulase-negative *Staphylococci* were considered as contaminants of bacterial cultures. However, this group especially *S. epidermidis* has emerged as an important pathogen and a major cause of serious infections in ICU patients [1, 20]. In this study, *S. epidermidis* was the most common isolated member of CoNS followed by *S. capitis*. This was in agreement with prior reports conducted in adult ICUs [5, 20].

In the current study, resistance of CoNS to oxacillin was 76.6%. Our study supports previous reports that show increased resistance of CoNS to oxacillin in which oxacillin resistance among CoNS reached 82.4% [21]. Owing to increased resistance to methicillin among CoNS, vancomycin is frequently considered as the first choice in antimicrobial therapy [22]. In contrast to oxacillin, all CoNS isolates in the present study were sensitive to vancomycin. This was in agreement with other studies conducted on different samples demonstrated that all isolates of CoNS were sensitive to vancomycin [23–25]. Vancomycin resistance is still infrequent in CoNS. However, heterogeneous resistance was reported among CoNS and was associated with failure of vancomycin therapy. These heteroresistant microcolonies may be a precursor of vancomycin resistance [5, 6]. These heterogeneous resistant strains have a susceptible vancomycin MIC, but they can grow in presence of 4 µg/mL vancomycin which is greater than their MIC [19].

Despite the presence of many methods for diagnosis of vancomycin heteroresistance in *Staphylococci* as modified E test, PAP, PAP-AUC and disc diffusion method [26–28], still PAP method is the most reliable method and considered as the gold standard for the detection of heteroresistant *Staphylococci* [29]. In the present study, nine isolates (15.5%) showed vancomycin heteroresistance profile identified by growth on BHI containing 4 µg/mL vancomycin and confirmed by PAP. The prevalence of heteroresistance was similar to previous reports that range from (7.4 to 18.3%) [28, 21]. All heteroresistant isolates were oxacillin resistant and most of them were associated previous vancomycin therapy. In spite of susceptibility of all isolates to vancomycin, there was an increase in vancomycin MIC range of oxacillin resistant isolates (.25–2 µg/mL) towards the intermediate cutoff. In prior studies, increase in vancomycin MIC and rise of heteroresistant *Staphylococci* among BSI was attributed to previous use of β lactams or glycopeptides, patients admission to ICU and high rate of oxacillin resistance [28, 4]. Many previous reports show the possible relation of reduced susceptibility to glycopeptides and methicillin resistant CoNS isolates [30]. More studies extending longer durations are needed to investigate if there is a gradual increase in vancomcin MICs in oxacillin resistant CoNS like vancomycin MICs creep in methicillin resistant *S. aureus*.

## Conclusion

This study alerts about the emergence of oxacillin-resistant CoNS showed heteroresistance to vancomycin. These heteroresistance strains were susceptible to vancomycin and cannot be detected by conventional standard methods. This implies the need of screening test for heteroresistance to avoid therapeutic failure. So, we should be aware of the potential reduction in vancomycin susceptibility of this pathogen, and this should be considered in determination of both empirical and rational therapy.

## Abbreviations

CoNS: coagulase negative *Staphylococci*
BSIs: bloodstream infections
ICUs: intensive care units
BHI: brain heart infusion
PAP: population analysis profile
